# Collagen I promotes hepatocellular carcinoma cell proliferation by regulating integrin β1/FAK signaling pathway in nonalcoholic fatty liver

**DOI:** 10.18632/oncotarget.21525

**Published:** 2017-10-05

**Authors:** Xinglong Zheng, Wenyan Liu, Junxi Xiang, Peng Liu, Mengyun Ke, Bo Wang, Rongqian Wu, Yi Lv

**Affiliations:** ^1^ Shaanxi Provincial Center for Regenerative Medicine and Surgical Engineering, Institute of Advanced Surgical Technology and Engineering, First Affiliated Hospital of Xi’an Jiaotong University, Xi’an, Shaanxi, China; ^2^ Department of Cardiovascular Surgery, First Affiliated Hospital of Xi’an Jiaotong University, Xi’an, Shaanxi, China; ^3^ Department of Hepatobiliary Surgery, First Affiliated Hospital of Xi’an Jiaotong University, Xi’an, Shaanxi, China

**Keywords:** nonalcoholic fatty liver, decellularized liver matrix, collagen I, integrin β1, hepatocellular carcinoma

## Abstract

Nonalcoholic fatty liver disease (NAFLD) has become a major risk factor for hepatocellular carcinoma (HCC) worldwide. However, the underlying mechanism remains insufficiently elucidated. The expression of Collagen I, an important component of extracellular matrix (ECM), was increased during the progression from simple steatosis to NASH. The purpose of this study was to investigate the role of Collagen I in NAFLD-related HCC. To study this, the decellularized liver matrix, which preserves the pathological changes of ECM, was prepared from the human fatty liver (FLM) and human normal liver (NLM). HepG2 cells cultured in FLM had a higher proliferation rate than those in NLM. SMMC-7721 and HepG2 cells cultured on Collagen I-coated plates grew faster than those on either Collagen IV- or fibronectin-coated plates. This effect was dose-dependent and associated with elevated integrin β1 expression and activation of downstream phospho-FAK. Knocking down the expression of integrin β1 significantly decreased the proliferation of HCC cells. Additionally, an orthotopic tumor model was established in NAFLD mice at different stages. The over-expressed Collagen I in the mice liver increased the expression of integrin β1 and downstream phospho-FAK, resulting in the proliferation of HCC cells. This proliferation could be inhibited by blocking the integrin β1/FAK pathway. In summary, our study demonstrated that Collagen I promoted HCC cell proliferation by regulating the integrin β1/FAK pathway. Decellularized liver matrix can be used as a platform to three-dimensionally culture HCC cells and reproduce the impact of changed ECM on the progression of NAFLD-related HCC.

## INTRODUCTION

With the rising obesity rate, the global prevalence of nonalcoholic fatty liver disease (NAFLD) has reached 25.24% [1]. NAFLD is now the most common liver disorder worldwide [2, 3]. As a progressive liver disease, NAFLD rangs from simple steatosis to nonalcoholic steatohepatitis (NASH) and eventually cirrhosis. NAFLD is a major risk factor for hepatocellular carcinoma (HCC) [4–6]. The HCC incidence increases from 0.44/1000 person-years in NAFLD patients to 5.29/1000 person-years in NASH patients [1]. However, the underlying mechanism remains insufficiently elucidated.

The extracellular matrix (ECM), a three-dimensional structure with distinct biochemical and biomechanical properties, is an important component of tumor microenvironment. Changes in the composition or architecture of the ECM have been shown to promote tumor growth, angiogenesis and metastatic progression [7, 8]. Integrin β1, a transmembrane receptor, mediates cell-ECM communication [9, 10]. It can activate multiple intracellular pathways including the FAK pathway to regulate cell proliferation, migration, invasion and survival [11, 12]. The progression from simple steatosis to NASH is associated with profound changes in the ECM. Accumulation of fat in the liver creates a proinflammatory milieu, which in turn leads to the activation of hepatic stellate cells (HSCs), resulting in massive collagen deposition in NASH patients [13]. Collagen I is a major component of the liver ECM and significantly upregulated at the stage of NASH. Koenig [14] found Collagen I can induce disruption of E-cadherin mediated cell-cell contacts and promote proliferation of pancreatic carcinoma cells. However, whether the increased risk of carcinogenesis in NASH patients is associated with the accumulation of Collagen I in the liver remained unclear. We therefore hypothesized that upregulation of Collagen I in NASH promotes HCC cell proliferation by regulating the integrin β1/FAK signaling pathway.

The decellularization process removes cells from an organ but preserve its 3D ultrastructure, which consists of the natural ECM [15]. Decellularized matrix has been widely used in organ reconstruction [16–18]. Recently, more and more attention has been paid to the decellularized matrix in tumor research, as it provides an excellent 3D cell culture environment [19–21]. The decellularized matrix preserves the pathological changes of ECM and simulates the *in vivo* tumor microenvironment. To investigate the effect of increased Collagen I on NASH-related tumor proliferation, we used the decellularized matrix of human nonalcoholic fatty liver to reproduce the NASH microenvironment *in vitro*, and then verified the findings by using the 2D cell culture model and two mouse models of NAFLD/NASH. We found that Collagen I promotes HCC cell proliferation by regulating the integrin β1/FAK signaling pathway in nonalcoholic fatty liver.

## RESULTS

### Nonalcoholic fatty liver promotes hepatocellular carcinoma cell proliferation

**Figure 1:**
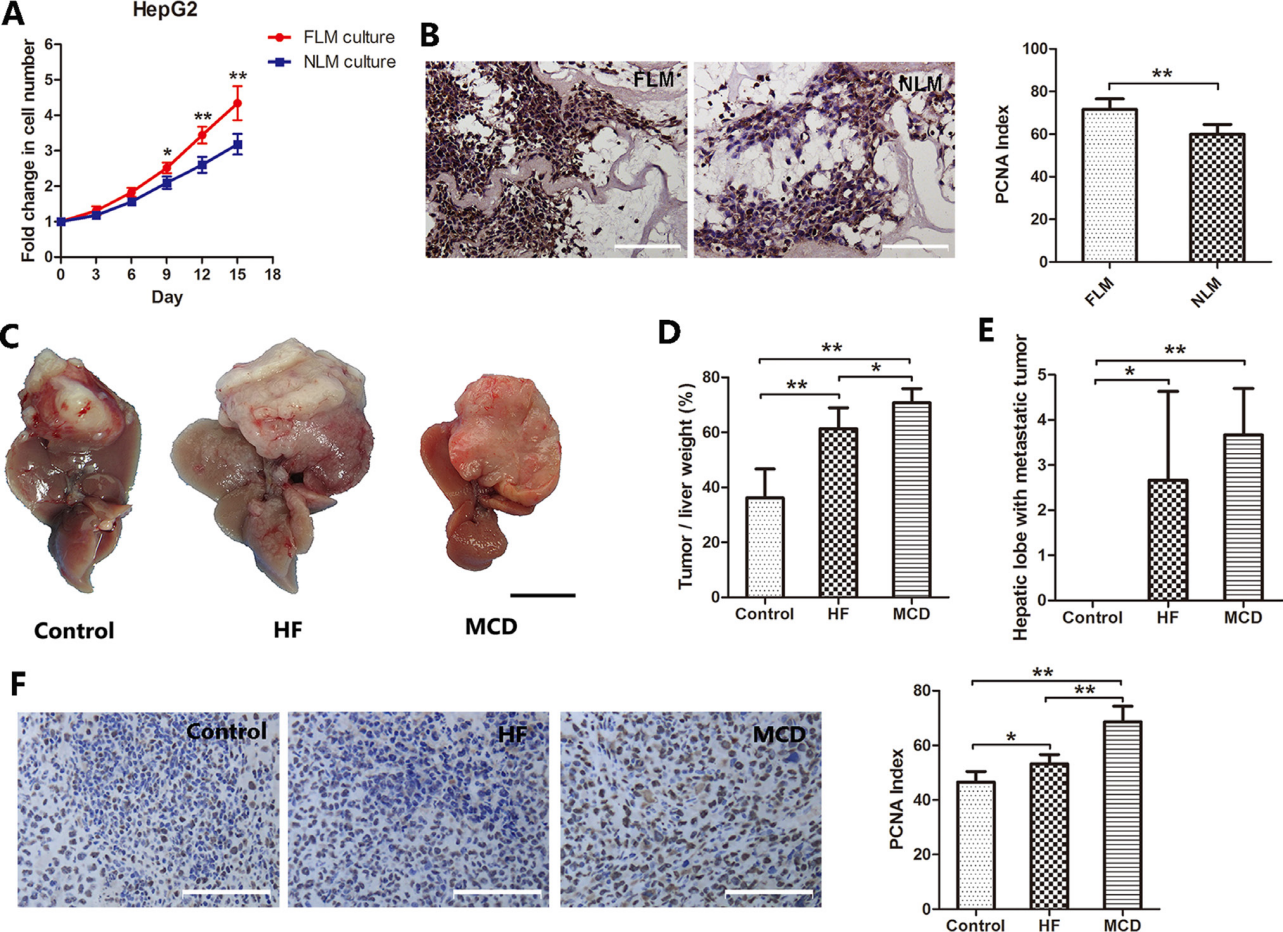
Nonalcoholic fatty liver promotes HCC cells proliferation (**A**, **B**) Cell proliferation profiles of HepG2 cells cultured in human fatty liver matrix (FLM) and human normal liver matrix (NLM). HepG2 cells were seeded into the NLM and FLM and cultured for 15 days. The proliferation of HepG2 cells were measured by residual DNA quantification. (A) HepG2 cells grew faster in FLM than NLM after day 9 (*n* = 6). (B) PCNA staining and PCNA index of HepG2 cells cultured in the FLM and NLM. (**C**–**F**) HCC growth in NAFLD/NASH mice. Mouse models of NFALD/NASH were induced by feeding the mice with either a high fat diet (HF) or a methionine–choline-deficient diet (MCD). Control mice were fed a standard commercial mouse diet (Control). Twelve weeks later, 5 × 10^6^ H22 cells (a mouse liver cancer cell line) were injected into the mouse liver. Two weeks after injecting H22 cells, the mice were sacrificed. The tumor growth in the liver was assessed. (C) Representative macroscopic liver tumor images of each group. (D) The percentage of tumor weight account for liver weight (*n* = 6). (E) The number of hepatic lobes with metastatic tumor (*n* = 6). (F) PCNA staining and PCNA index of liver tumor. Scale bar: 100μm (B, F); 1 cm (C), *P < 0.05, **P < 0.001.

The human fatty liver had severe steatosis and was determined unsuitable for transplantation. The normal donor liver was discarded due to a long warm ischemia-induced damage. Decellularized liver matrix preserves the sophisticated assembly of liver ECM. It provides an excellent three-dimensional cell culture environment for tumor studies. To study the role of ECM in fatty liver-associated HCC, decellularized liver matrices were prepared from human nonalcoholic fatty livers (fatty liver matrix, FLM) and a human normal liver (normal liver matrix, NLM) as we described before (Supplementary Data). HepG2 cells were seeded into the NLM and FLM and cultured for 15 days. The proliferation of HepG2 cells were measured by residual DNA quantification. As shown in Figure 1A, HepG2 cells displayed a significantly faster proliferation rate in the FLM than in the NLM. At 15 days after the inoculation, the DNA content in the FLM culture was 36.4% more than those in the NLM culture (*P* < 0.001). Similarly, the PCNA index, an indicator of cell proliferation, was 19.7% higher in cells cultured in the FLM than those in the NLM (*P* < 0.001, Figure 1B). These results show that the ECM of the nonalcoholic fatty liver could promote HepG2 cell proliferation compared to the ECM of the normal liver.

To confirm this finding *in vivo*, we constructed the mouse models of NAFLD and NASH, and injected H22 cells into the liver to observe the difference of tumor growth *in vivo* (supplementary data). Two weeks after injecting H22 cells, the tumor tissue was obviously seen on the liver lobe. As shown in Figure 1C, the tumor tissue in the HF and MCD groups almost occupied the whole left lateral lobe, and the left lobe became compensatory hyperplasia. The tumor weight/liver weight percentage in the control, HF and MCD groups were 36.2±10.5%, 61.3±7.7% and 70.7±5.2%, respectively. The differences were statistically significant between the groups (Figure 1D). Furthermore, intrahepatic metastasis of the tumor was found in the HF and MCD groups, but not in the control group (Figure 1E). There were also significantly more PCNA positive cells in mice fed with either HF or MCD diet than those with control diet (Figure 1F).

Taken together, these results indicate that nonalcoholic fatty liver promotes HCC cell proliferation and the ECM plays an important role in it.

### Collagen I contributes to the NAFLD-related HCC

**Figure 2:**
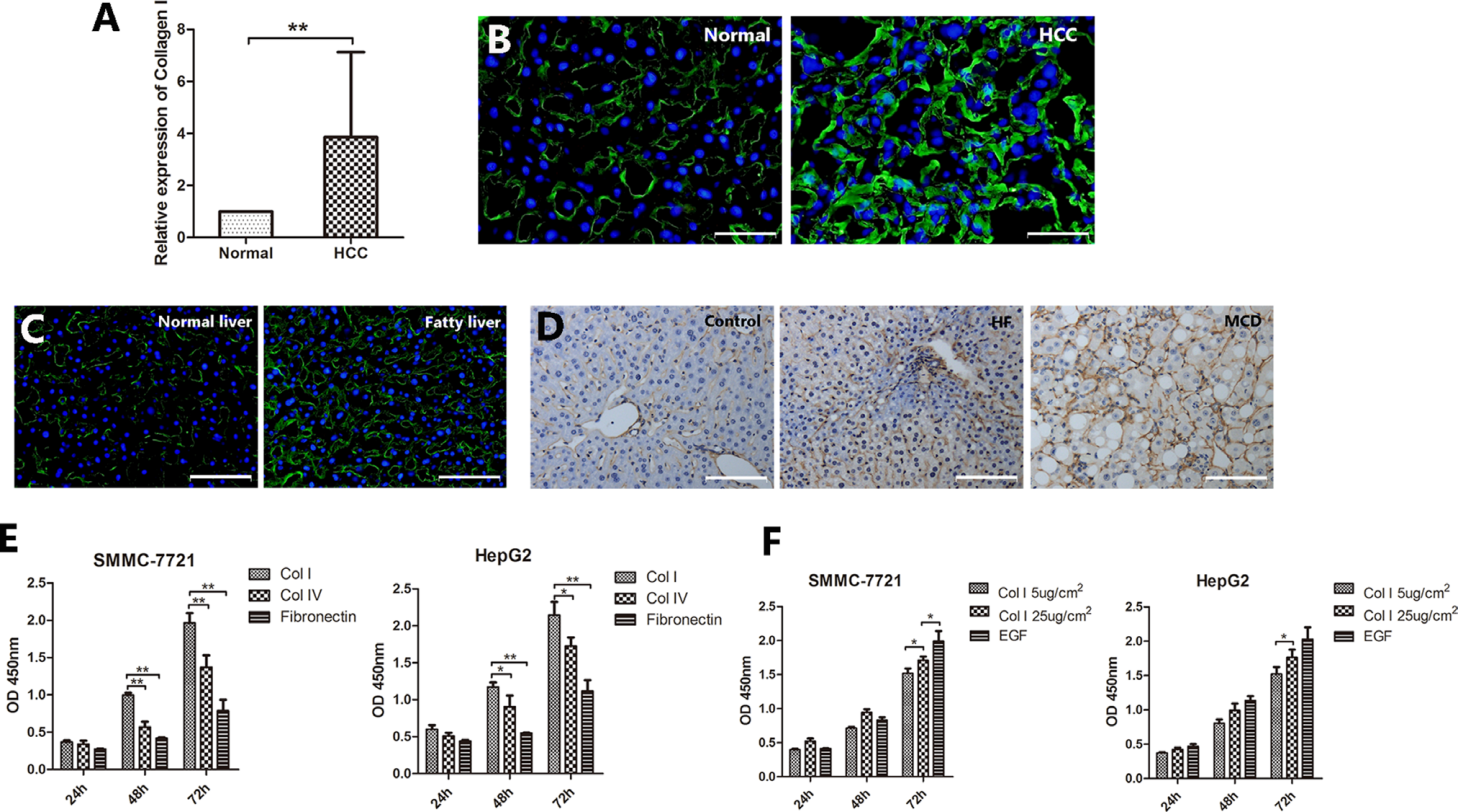
Collagen I contributes to the NAFLD-related HCC (**A**) qRT-PCR analysis showed that Collagen I levels were 2.8 fold higher in HCC samples than those in adjacent non-tumor liver tissues. (**B**) Immunofluorescence staining confirmed the higher expression of Collagen I (green) in HCC tissues compared to normal liver tissues. (**C**) Immunofluorescence staining revealed a higher expression of Collagen I (green) in human fatty liver compared to human normal liver. (**D**) In the mouse models of NAFLD/NASH, Collagen I was found to be upregulated. (**E**, **F**) HCC cells cultured on different ECM or different concentration of Collagen I to determine the effect of Collagen I on HCC cell proliferation. The vitality of cells at each time point was detected by CCK-8 assay. (E) SMMC-7721 and HepG2 cells cultured on Collagen I proliferated significantly faster than those on either Collagen IV or fibronectin. (F) The effect of Collagen I on cell proliferation appeared to be dose-dependent. Scale bar: 50 μm (B); 100 μm (C, D), *P < 0.05, **P < 0.001.

Collagen I is an important component of the liver ECM. Collagen I expression was up-regulated in 83.7% of human HCC specimens compared with adjacent non-tumor tissue. qRT-PCR analysis showed that Collagen I levels were 2.8 fold higher in HCC samples than those in normal liver samples (*P* < 0.001, Figure 2A). This result was confirmed by immunofluorescent staining of Collagen I in HCC and normal liver tissues (Figure 2B). Interestingly, the expression of Collagen I was also increased in the human fatty liver compared to the normal liver (Figure 2C). In the mouse models of NAFLD/NASH, Collagen I was also found to be upregulated (Figure 2D).

To determine the effect of Collagen I on HCC cell proliferation, SMMC-7721 and HepG2 cells were cultured on Collagen I, Collagen IV and fibronectin, respectively, for 3 days. As shown in Figure 2E, cells cultured on Collagen I proliferated significantly faster than those on either Collagen IV or fibronectin (*P* < 0.001). The effect of Collagen I on cell proliferation appeared to be dose-dependent (Figure 2F).

Taken together, these results indicate that Collagen I is overexpressed in both HCC and NAFLD and it is associated with increased HCC cell proliferation.

### Integrin β1/FAK signaling pathway is involved in NAFLD-related HCC

**Figure 3:**
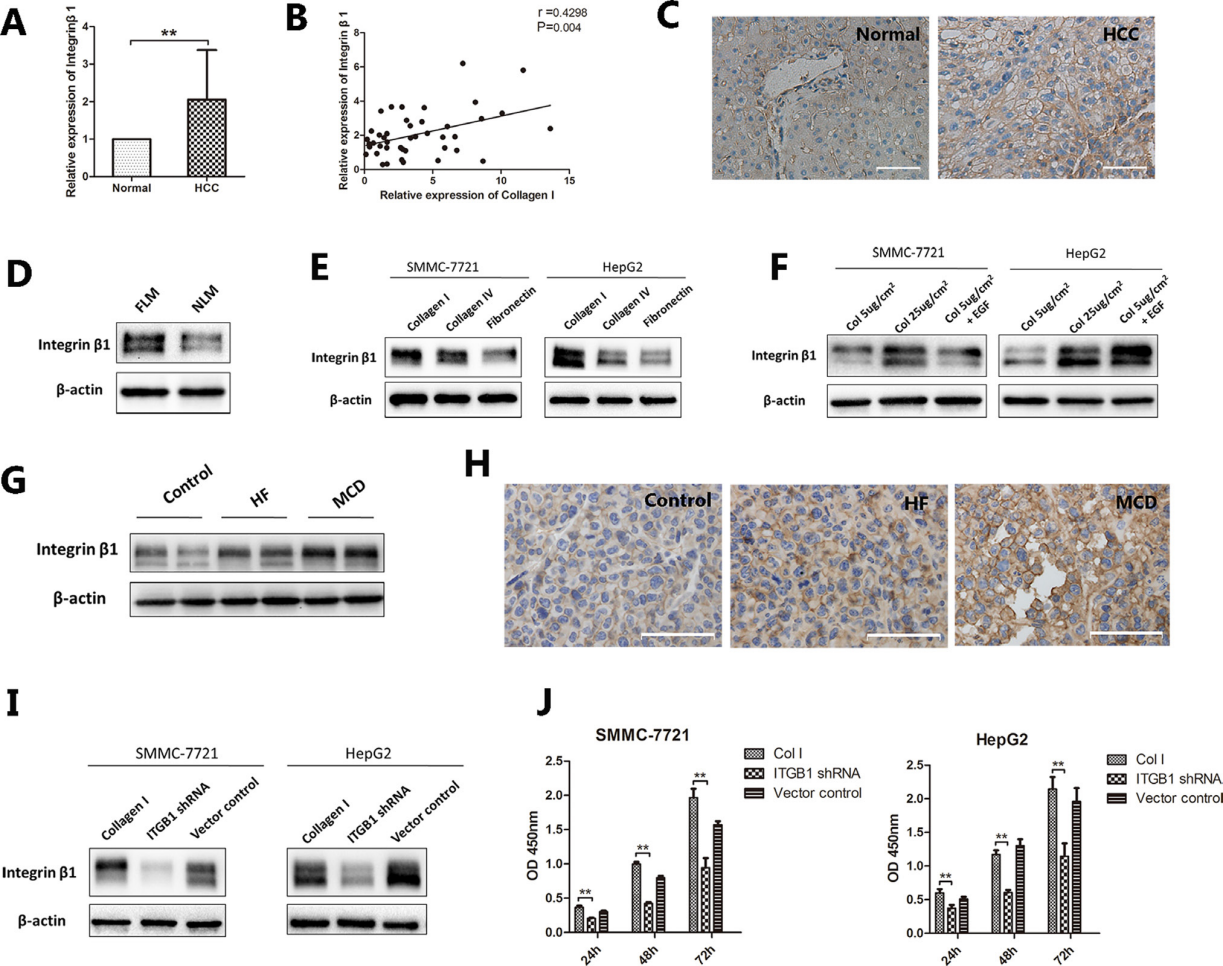
The expression of integrin β1 in NAFLD-related HCC (**A**–**C**) The expression of integrin β1 in HCC. (A) qRT-PCR analysis showed that integrin β1 levels were 1.1 fold higher in HCC samples than those in adjacent non-tumor liver tissues. (B) Correlation analysis indicated that the expression level of integrin β1 were positively correlated with the level of Collagen I, r = 0.43, *P* = 0.004. (C) Immunohistochemical staining revealed a high expression of integrin β1 in HCC tissues. (**D**) HepG2 cells were seeded into the NLM and FLM and cultured for 15 days. Western blot indicated that cells cultured in FLM had a higher expression of integrin β1 than those in NLM. (**E**, **F**) HCC cells cultured on different ECM or different concentration of Collagen I. (E) For SMMC-7721 and HepG2, integrin β1 was higher expressed in Collagen I compared to Collagen IV and fibronectin. (F) Increasing Collagen I concentration could up regulated the expression of integrin β1 in both cell lines. (**G**, **H**) The expression of integrin β1 in mouse orthotopic tumor model. (G) Western blot indicated that tumors grown in HF- or MCD-fed mice expressed higher level of integrin β1. (H) Immunohistochemical staining of integrin β1 confirmed the same results. (**I**–**J**) Integrin β1 was knocked down in HCC cells by shRNA technology through lentiviral transduction. (I) Western blot confirmed the stable knock-down of integrin β1 in both cell lines. (J) HepG2 and SMMC-7721 cells cultured on Collagen I grew significantly slowly after knocking down integrin β1. Scale bar: 100 μm (C), 50 μm (H), *P < 0.05, **P < 0.001.

Integrin β1 is a transmembrane receptor. It plays a vital role in cell-ECM interactions. The expression levels of integrin β1 were 1.1 fold higher in HCC tissue than those in adjacent non-tumor tissue (*P* < 0.001, Figure 3A). Correlation analysis showed that the expression level of integrin β1 was positively correlated with the level of Collagen I (r = 0.43, *P* < 0.05, Figure 3B). Immunohistochemical staining also comfirmed the higher expression of integrin β1 in HCC compared with normal liver tissues (Figure 3C). In addition, HepG2 cells cultured in the FLM had a higher expression of integrin β1 than those in the NLM (Figure 3D). Similarly, SMMC-7721 and HepG2 cells cultured on Collagen I expressed higher levels of integrin β1 than those on Collagen IV or fibronectin (Figure 3E). And the effect of Collagen I on integrin β1 expression was dose-dependent in both cell lines (Figure 3F). Tumors grown in HF- or MCD-fed mice also expressed higher levels of integrin β1 than those in control diet-fed mice (Figure 3G, 3H). To determine the contribution of integrin β1 in NAFLD-related HCC, integrin β1 was knocked down in HepG2 and SMMC-7721 cells by the shRNA technology through lentiviral transduction. The stable knock-down of integrin β1 was confirmed by Western blotting (Figure 3I). As shown in Figure 3J, both HepG2 and SMMC-7721 cells cultured on Collagen I grew significantly slowly after knocking down the expression of integrin β1 (*P* < 0.001), while control vectors had little effects on cell proliferation.

**Figure 4:**
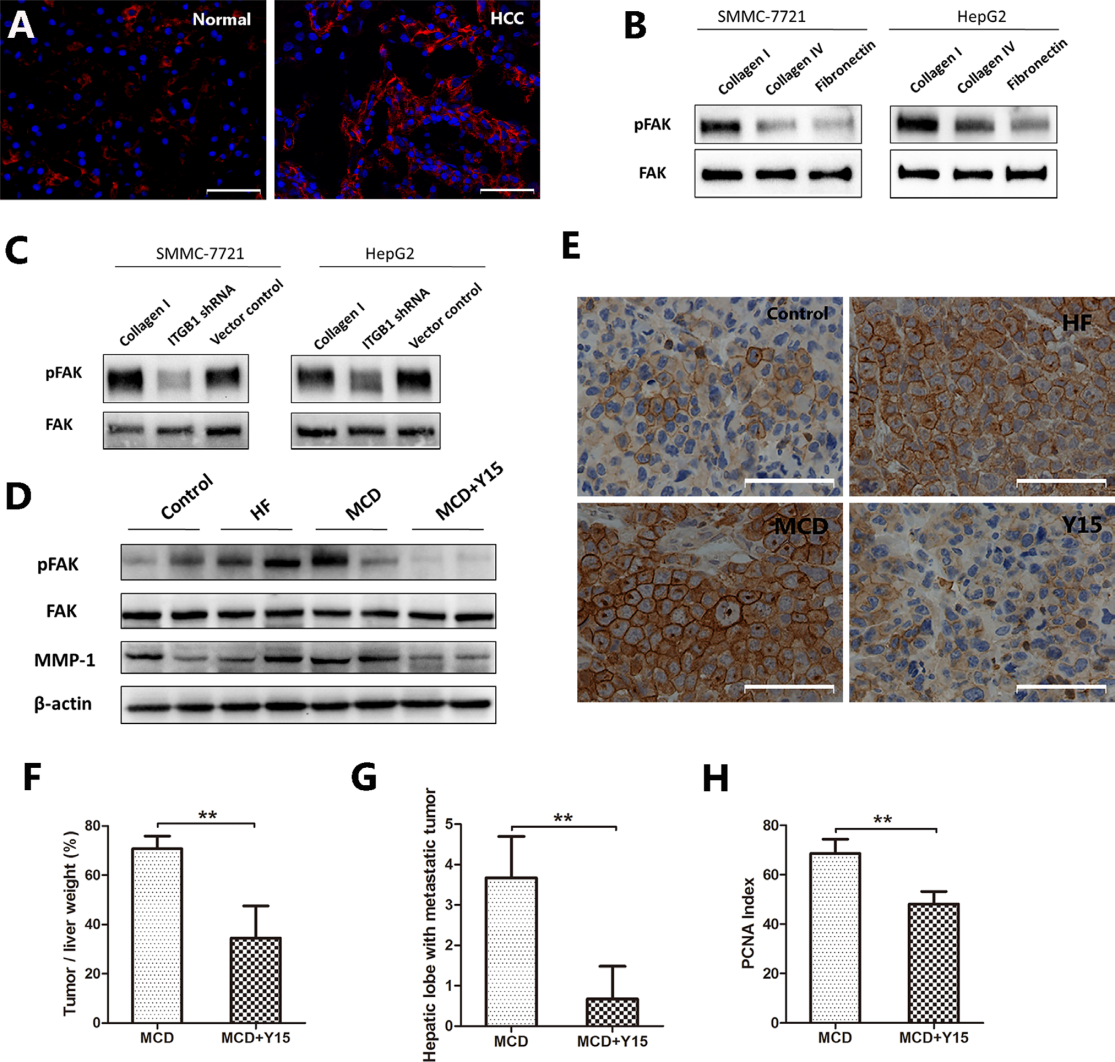
The expression of phospho-FAK in NAFLD-related HCC (**A**) Immunofluorescence staining revealed the high expression of phospho-FAK (red) in HCC tissues. (**B**) Phospho-FAK was higher expressed in the HCC cells cultured on Collagen I compared to Collagen IV and fibronectin. (**C**) Phospho-FAK was low expressed after integrin β1 knocking down in both cell lines. (**D**–**H**) In mouse orthotopic tumor model, a specific inhibitor of FAK, Y15, was used in MCD-fed mice. (D) Western blot indicated that tumors grown in HF- or MCD-fed mice expressed higher levels of phospho-FAK and MMP-1 than those in control diet-fed mice, while Y15 significantly decreased the expression of phospho-FAK and MMP-1 in the tumors of MCD-fed mice. (E) Immunohistochemical staining confirmed the same results. (F) Liver tumor grew smaller after inhibiting the expression of phospho-FAK. (G) Decreased intrahepatic metastasis after inhibiting the expression of phospho-FAK. (H) There was decreased number of PCNA positive cells after inhibiting the expression of phospho-FAK. Scale bar: 50 μm (A, E), *P < 0.05, **P < 0.001.

FAK is a downstream molecule connecting integrin β1. Compared with normal liver tissues, HCC tissues expressed higher levels of Phospho-FAK (Figure 4A). SMMC-7721 and HepG2 cells cultured on Collagen I also showed enhanced expression of phospho-FAK compared to Collagen IV and fibronectin (Figure 4B). The expression of phospho-FAK was significantly decreased after knocking down integrin β1 in both SMMC-7721 and HepG2 cells (Figure 4C). In the mouse models, tumors grown in HF- or MCD-fed mice also expressed higher levels of phosphor-FAK than those in control diet-fed mice. Adminstration of Y15, a specific inhibitor of FAK, significantly decreased the expression of phospho-FAK (Figure 4D, 4E). At the same time, the tumor weight/liver weight percentage of MCD-fed mice were reduced by more than 50% after Y15 treatment (*P* < 0.001, Figure 4F). Furthermore, the numbers of hepatic lobes with metastatic tumors and the PCNA index were significantly decreased by Y15 (Figure 4G, 4H). Besides, matrix metalloproteinase-1 (MMP-1), one of the main enzymes involved in ECM degradation, was increased expressed in the tumor tissues of HF- and MCD-fed mice. However, the expression of MMP-1 was significantly decreased after administration of Y15. This result was consistent with the trend of intrahepatic metastasis.

Taken together, these results suggest that the integrin β1/FAK signaling pathway plays an important role in NAFLD-related HCC.

## DISCUSSION

In the present study, we used decellularized liver matrix, FLM and NLM, to reproduce the effect of ECM changes in nonalcoholic fatty liver on HCC cell proliferation. The traditional two-dimensional culture lacks the interaction between cells and ECM, which leads to a decreased malignant degree and significant changes in differentiation, polarity and intercellular communication for tumor cells [22, 23]. The decellularized human liver matrix not only provided a three-dimensional environment more close to the *in vivo* conditions, but also retained the difference of ECM between the NAFLD and normal liver. The faster growth and higher PCNA index of HepG2 in FLM compared to NLM indicated that the ECM of nonalcoholic fatty liver might promote the proliferation of HCC.

Decellularized liver matrix is a complex mixture of various structural and functional proteins [16]. Collagen I, Collagen IV and fibronectin are three most commom kinds of ECM proteins. To determine which component of ECM is responsible for NAFLD-related HCC, we cultured SMMC-7721 and HepG2 cells on plates coated with collagen I, collagen IV or fibronectin. This experiment showed that HCC cells cultured on Collagen I had a higher proliferation rate than those on Collagen IV and fibronectin, which is associated with a higher expression of integrin β1. Thus, Collagen I might be an activator of integrin β1 and play a more important role in cell proliferation compared to Collagen IV and fibronectin. Furthermore, we found both Collagen I and integrin β1 were upregulated in human HCC specimens. A reasonable correlation coefficient was found between collagen I and integrin β1 in HCC. Integrin β1 is the key transmembrane protein that conducts extracellular biochemical and biomechanical signals into the cell. Several studies demonstrated that matrix stiffness contributes to the proliferation, development, and chemoresistance of tumor cells through FAK, PKB/AKT, and PTEN pathways [24, 25]. In this study, we found that knocking down the expression of integrin β1 in HCC cells could reduce proliferation and inhibit downstream pathway activation. Moreover, the over-expressed Collagen I in the liver of NAFLD/NASH mice were associated with increased expression of integrin β1 and downstream phospho-FAK, resulting in the proliferation of H22 cells. This proliferation could be inhibited by blocking the integrin β1/FAK pathway. On the other hand, inhibiting the expression of phospho-FAK could significantly reduce the degradation of ECM by tumor cells, and then reduce the intrahepatic metastasis. These results indicate that the over-expressed Collagen I in nonalcoholic fatty liver could promote HCC cell proliferation by regulating the integrin β1/ FAK pathway.

It is noteworthy that more than one third of patients with NAFLD-related HCC in the clinical had non-cirrhotic NAFLD [26]. A cross-sectional study from Japan analyzed 87 cases of HCC patients with histologically proven NASH and found 43 cases do not develop into cirrhosis [27]. Thus, hepatic steatosis alone may contribute to the development of HCC. Similarly, in our study, the expression of Collagen I only increased moderately in the HF group compared to the control group, but the volume of tumor in the HF group was significantly higher than that of the control group. It can be speculated that the accumulation of lipid droplets and the associated inflammatory responses may also contribute to the progression of HCC. Ma [28] found NAFLD causes selective CD4^+^T lymphocyte loss due to its high level of mitochondrially derived reactive oxygen species in fatty acid metabolism, and the impaired anti-tumor surveillance promotes hepatocarcinogenesis. In addition, the procarcinogenic environment related to epigenetic and genetic events [5], sustained proliferative signaling providing by hyperinsulinism [29] and soluble endocrine mediators derived from gut microbiome [30, 31] are all implicated in the progression of NAFLD-related HCC. Thus, multiple mechanisms drive tumorigenesis and HCC development in NAFLD.

## MATERIALS AND METHODS

### Human tissue specimens and liver decellularization

43 paired cryptogenic hepatocellular carcinoma and adjacent non-tumor liver tissues were collected from patients underwent resection at First Affiliated Hospital of Xi’an Jiaotong University. Those tissues were used for qRT-PCR analysis. Three fatty livers diagnosed as severe macrovesicular steatosis (diagnosed as nonalcoholic fatty liver according to their medical history) and one normal liver were collected from clinical inapplicable DCD organs. The human organs used in this study were obtained under standard procurement protocols from the New England Organ Bank and in accordance with the Declaration of Helsinki. The study was approved by the Ethics Committee of Xi’an Jiaotong University and informed consent was obtained from all patients and voluntary participation prior to the study. The process of liver decellularization is described in the supplementary data.

### 3D cultures on scaffolds

The NLM and FLM were further cut into 5×5x3mm pieces, and placed in 6-well culture plates, incubated with medium overnight. Approximately 5×10^5^ HepG2 cells were seeded into NLM and FLM. The scaffolds were placed in a cell incubator (Steri-Cycle 371,Thermo, USA) at 37°C and 5% CO_2_ for 30 min to allow cells adhere. Then another 3ml culture medium was added into each well for further culture. The scaffolds were cultured for 3d, 6d, 9d, 12d and 15d respectively (*n* = 6). The medium contains Dulbecco's Modified Eagle Medium (DMEM, Gibco, USA), 10% fetal bovine serum (FBS, Gibco), 100 U/mL penicillin, and 100 mg/L streptomycin. Medium was changed every other day.

### DNA quantification

The proliferation curves of HepG2 were drawn by comparing the changes of DNA quantification in NLM and FLM. The recellularized NLM and FLM (*n* = 6) were placed in centrifuge tubes, lyophilized later, and then DNA was isolated by using Genomic DNA Kit (TIANGEN Biotech, China) according to the manufacturer's instructions. Briefly, samples were digested with DNA isolation solutions. Proteinase K digestion was used in the procedure and tubes containing the tissue samples were vortexed for 10 minutes and incubated at 56°C for 30 minutes. After the protein was precipitated and discarded, the remaining DNA was diluted with 1mL TE buffer (10 mM Tris-HCl, 1mM EDTA, pH 7.5). The total amount of DNA was quantified using Varioskan Flash (Thermo).

### NAFLD/NASH model and orthotopic tumor model

All the animal work performed was in accordance with the animal welfare act, institutional guidelines and was approved by the institutional animal care and use committee of Xi’an Jiaotong University. Male C57BL/6J mice, aged 8 weeks, after adaptive diet for one week, were fed a high fat (HF) diet with 60 kcal% (Research Diets D12492) or a methionine–choline-deficient (MCD) diet (Trophic Animal Feed High-Tech Co. Ltd, China) up to 12 weeks to induce NAFLD/NASH model. Control mice were fed a standard diet. For orthotopic tumor model, the mice were anesthetized by isoflurane, followed by a median incision. After exposing the left lateral lobe, 50 μl cell suspension containing 5×10^6^ H22 cells (mouse liver cancer cell line) was injected into the liver, sealed with biological medical glue (compont, China). Another tumor group of NASH model was treated with FAK inhibitor Y-15 (MedChem Express, MCE, USA) by intraperitoneal injection, at a concentration of 30mg/Kg daily 5days/week. Two weeks later, the mice were sacrificed and the samples were obtained. The weight of each liver and tumor and the number of the hepatic lobes containing metastatic tumor were recorded.

### Histological evaluation

To evaluate NAFLD/NASH model, fibrosis was assessed using Masson's trichrome staining. The activated hepatic stellate cells (HSCs) were indicated by α-smooth muscle actin staining (α-SMA). Lipid accumulation was detected by Oil Red O staining in frozen liver sections. The liver histology was evaluated by an expert pathologist who was blinded to the intervention condition. Histology was assessed using the NAFLD activity score (NAS) and fibrosis score [32] (Supplementary data). The PCNA index was quantified by counting the PCNA positive cells for 6 independent samples on 5 images per sample by the same experimenter blind to the condition. The mean value of the single sample was then used for statistical evaluation.

### Quantitative RT-PCR

RNA was extracted from fresh frozen HCC tissues and human liver cancer cell lines (HepG2 and SMMC-7721) by using Trizol solution (Invitrogen). The sample absorbance at 280 nm and 260 nm was measured using Varioskan Flash to confirm RNA concentration and quality. Reverse transcription was performed using PrimeScript™ RT reagent kit (Takara) following the manufacturer's recommendation. qRT-PCR analysis was performed for ITGB1 by using the StepOnePlus™ Real-Time PCR System (Applied Biosystems). The alteration in gene expression was obtained using the ΔΔCt method in which all samples were first normalized against the level of glyceraldehyde 3-phosphate dehydrogenase (GAPDH) present in the same sample. qRT- PCR analysis was repeated in triplicate. The primers were as follows, ITGB1-F: 5′-GTAACCAACCGTAGCAAAGGA-3′, Collagen I-F: 5′-CAATGGTGCTCCTGGTATT-3′ GAPDH-F: 5′-TGACTTCAACAGCGACACCCA-3′; ITGB1-R: 5′-TCCCCTGATCTTAATCGCAAAAC-3′, Collagen I-R: 5′-CTCGCTTTCCTTCCTCTC-3′, GAPDH-R: 5′-CACCCTGTTGCTGTAGCCAAA-3′.

### RNA interference assay

Three lentiviral shRNA vectors targeting human ITGB1 gene and a control vector were purchased from Shanghai GeneChem, Co. Ltd, China. Lentivirus was packed in HEK293T cells. The three kinds of viral supernatant was respectively added into HepG2 cells which have a higher expression of integrin β1 compared to SMMC-7721 (at multiplicity of infection = 10) with ENi.S and 5 μg/ml polybrene. The optimal sequence against human ITGB1 was 5′- CTTGCATTACTGCTGATAT -3′. Then the HCC cells were infected with this lentivirus to obtain stably-infected HCC cells with integrin β1 knock-down. Efficiency of shRNAs was confirmed by qRT-PCR and western blotting.

### Cell culture on ECM

Human HCC cell lines HepG2 and SMMC-7721 were cultured in DMEM and 1640 medium supplemented with 10% FBS, 100U/mL penicillin, and 100 mg/L streptomycin. To compare the effects of different ECM on the proliferation of hepatocellular carcinoma cells, 10^5^ HepG2 and SMMC-7721 cells were plated on 6-well culture plate pre-coated with 5 ug/cm^2^ of either collagen I, collagen IV (BD Biosciences, Canada) or fibronectin (Thermo Fisher scientific, USA) for 24 h. After reaching confluence, protein was extracted from these cells for western blotting. In order to further analyze the effects of different concentrations of collagen I on the proliferation, cells were also plated on 6-well culture plate pre-coated with 25ug/cm^2^ of collagen I or adding recombinant human epidermal growth factor (EGF, Peprotech, USA) to the medium at a concentration of 100 ng/mL.

### CCK-8 assay

The viability of HCC cells cultured on different ECM or adding EGF were quantitatively determined by the 2-(2-methoxy-4-nitriphenyl)-3-(4-nitrophenyl)-5-

(2,4-disulfophenyl)-2H-tetrazolium, monosodium salt (CCK-8) assay (Cell Counting Kit-8, Dojindo). Briefly, the 96-well culture plate was pre-coated with 5 ug/cm^2^ of either collagen I, collagen IV, fibronectin or 25 ug/cm^2^ of collagen I for 24 h. 100 μL medium containing 5 × 10^3^ cells was added onto the plate (*n* = 3). CCK-8 assay was performed at 24 h, 48 h and 72 h after cells plated. 10 μL of CCK-8 stock solution was added to each well and incubated at 37°C for 1.5 h. Optimal density (OD) value for absorbance was measured at a wavelength of 480 nm in Varioskan Flash.

### Western blotting

Equal amount of protein from each group was fractionated by 8% or 10% sodium dodecylsulfate- polyacrylamide gel electrophoresis (SDS-PAGE) and transferred onto a PVDF membrane (Millipore, Germany). Membranes were incubated with primary antibody overnight at 4°C, followed by horse radish peroxidase (HRP)-conjugated secondary antibodies. The antibodies used in this study were anti-integrin β1, anti-pFAK-Y397 (1:1000, Abcam), anti-FAK, anti-AKT, anti-pAKT-Ser473 (1:1000, CST), anti-MMP-1(1; 1000, proteintech, USA) and anti-β-actin (1:3000, proteintech). Proteins were detected using Clarity™ western ECL substrate (Bio-Rad Laboratories) and viewed by the imaging system (Universal hood III, Bio-Rad).

### Immunofluoresence

For immunofluorescence staining, samples were cryostat sectioned and placed upon glass slides. Samples were then rehydrated, permeabilized, blocked with 1% bovine serum albumin (BSA, Sigma) and treated with primary antibodies overnight at 4°C. The antibodies used in this study were anti-collagen I (1:100, Abcam) and anti-pFAK-Y397 (1:100, Abcam). Thereafter the samples were incubated at 37°C for 1 h with the secondary antibodies and counterstained with DAPI . Samples were imaged using Olympus BX53F.

### Statistical analysis

All data are expressed as the mean±SD (standard deviation). Statistical significance was determined by using SPSS 12.0 software (SPSS, San Rafael, CA, USA). Analysis of variance followed a Student's test was used to determine significant difference between the control and experimental group. The *P*-values less than 0.05 were considered significant.

## CONCLUSIONS

In summary, using decellularized liver matrix based 3-D cell culture, different ECM components coated 2-D cell culture and mouse models of NAFLD/NASH, we demonstrated that Collagen I promoted HCC cell proliferation by regulating the integrin β1/FAK pathway. Decellularized liver matrix, FLM and NLM, can be used as a platform to three-dimensionally culture HCC cells and reproduce the impact of changed ECM on the progression of NAFLD-related HCC. Our results further emphasize the role of tissue microenvironment in modulating biological behavior of HCC and provide a promising target for preventing and treating NAFLD-related HCC.

## SUPPLEMENTARY MATERIALS FIGURES AND TABLES



## Abbreviations

NAFLD: nonalcoholic fatty liver disease
NASH: nonalcoholic steatohepatitis
HCC: hepatocellular carcinoma
ECM: extracellular matrix
FLM: human fatty liver matrix
NLM: human normal liver matrix
