# Supplementary material for: Collagen I promotes hepatocellular carcinoma cell proliferation by regulating integrin β1/FAK signaling pathway in nonalcoholic fatty liver
Source: Oncotarget. 2017 Oct 5;8(56):95586–95. doi: 10.18632/oncotarget.21525 (PMC5707045; doi:10.18632/oncotarget.21525)
Supplement: Supplementary file 1 [file oncotarget-08-95586-s001.pdf]

Collagen I promotes hepatocellular carcinoma cell proliferation by regulating integrin β1/FAK signaling pathway in nonalcoholic fatty liver

SUPPLEMENTARY MATERIALS

RESULTS

Identification of decellularized matrix

After the decellularization procedure, the human liver turned khaki to translucent, and retained the complete shape of the liver. HE staining showed the absence of cellular components in NLM and FLM compared with the fresh liver (Supplementary Figure 1A). A lack of DAPI staining in NLM and FLM also confirmed the absence of cells (Supplementary Figure 1C). Immunohistochemical staining for three ECM proteins, Collagen I, Collagen IV and elastin indicated both the structural and basement membrane components of ECM were retained after decellularization (Supplementary Figure 1E). The ultrastructural characterization of the decellularized matrix was viewed by SEM images. The hepatocytes and other non-parenchymal cells existing in the liver originally had been completely removed, leaving a honeycomb structure (Supplementary Figure 1B). The residual DNA contents in the NLM and FLM were both less than 1% of that in the fresh tissue (Supplementary Figure 1D). These results suggested that the cell components were completely removed and the natural ECM was retained.

HepG2 cells cultured on NLM and FLM

Then we three-dimensional cultured HepG2 cells in NLM and FLM for 15 days. Histological staining of the recellularized FLM at 3d, 9d and 15d revealed the HepG2 cells distributed both in decellularized sinusoidal spaces and around the vessels and proliferated well (Supplementary Figure 2A). SEM showed HepG2 cells adhesion to the ECM of NLM and FLM in spherical or ellipsoidal shape (Supplementary Figure 2B).

Histological evaluation of NAFLD/NASH model

In order to further verify the changing Collagen I in the progression of NAFLD can promote the proliferation of HCC, male C57BL/6J mice were fed HF diet, MCD diet or normal diet for three months, following injecting H22 cells into the liver to observe the difference of tumor growth *in vivo* (Supplementary Figure 3A). Within 3 months of HF or MCD administration, the liver became tan and lighter in color compared to the control group, and liver volume got significantly smaller in MCD group. Liver histology indicated that the mice of HF group developed small droplet steatosis, while MCD group developed extensive macrovesicular steatosis, lobular inflammation and hepatocellular ballooning. Collagen fibrils could be seen in the sinusoids by Masson's trichrome staining and the activation of HSCs was further confirmed by α-SMA staining. It was obviously shown that massive collagen deposition and fibrogenesis were happened in MCD group (Supplementary Figure 3B). Histological score including NAFLD activity score and fibrosis score further demonstrated that the two models reproduced the different pathological states in the clinical progress of NAFLD (Supplementary Figure 3C, 3D). As activated HSCs play a role of cancer associated fibroblasts (CAFs) in the microenvironment of HCC, we examined the relationship between CAFs and Collagen I. As shown in Supplementary Figure 3E, the expression of α-SMA was increased in HF and MCD-fed mice compared with control mice, which was consistent with the expression of Collagen I.

MATERIALS AND METHODS

liver decellularization

The normal liver and fatty livers were frozen at −80°C for at least 24h and thawed at room temperature for 2 cycles. Decellularization was conducted by two peristaltic pumps at 100 mL/min and 50 mL/min respectively for portal vein and hepatic artery. The liver was initially perfused with deionized water for 30min, followed by 0.1% sodium dodecyl sulfate (SDS; MP Biomedicals, USA) for 48h, and 1% SDS for another 24 h. Subsequently, the liver was washed with deionized water for 30 min, 1% Triton X-100 (MP Biomedicals)/0.05%

EGTA (MP Biomedicals) for 24 h, and phosphate-buffered saline (PBS) for 6 h. Then the human normal liver decellularized matrix (NLM) was made, and it was cut into 2×2×1cm pieces. All cell components have been removed after these steps, but there were still a large number of lipid droplets in human fatty liver decellularized matrix (FLM). The FLM was also cut into the same size pieces, and 5–8 pieces were placed in one flask for processing. Briefly, samples were washed in deionized water for 2 h at room temperature with agitation (120 rpm), and then treated with 0.5 M NaCl for 3 h, 1 M NaCl for 3 h, and deionized water for 4 h in turn. The samples were treated with 0.25% trypsin (Amresco, Solon, OH)/ 0.05% EGTA at 37°C for 2 h and washing in deionized water for 1h, which were followed by treating with isopropanol overnight. After being treated with 1% Triton X-100 / 0.05% EGTA for 72 h (change the solution per 12h), the samples were rinsed in PBS for 24 h. Finally, these pieces of NLM and FLM were rinsed in 0.1% (v/v) peracetic acid / 4%EtOH for 2h, and then neutralized by PBS and deionized water washes for 1h each. The liver scaffolds were preserved in PBS containing 100 U/mL penicillin, 100 mg/L streptomyin (HyClone, Logan, UT) and kept at 4°C.

Morphologic and histological evaluation

Samples of fresh liver, decellularized liver and recellularized liver were fixed with 10% formalin, embedded in paraffin, and sectioned into 5 μm section for hematoxylin and eosin staining. The retention of extracellular matrix was analyzed by immunohistochemistry, anti-collagen I, anti-collagen IV and anti-elastin (1:100, Abcam, Cambridge, UK). 4’, 6-diamidino-2-phenylindole (DAPI, Sigma, St. Louis, MO) were used to stain nuclei of the fatty liver and FLM. For scanning electron microscopy (SEM), decellularized scaffolds and recellularized liver samples were fixed using 2.5% (v/v) glutaraldehyde in PBS. The samples were dehydrated by graded series of ethanol for 15 minutes each (50%, 75%, 80%, 95%, 100%), dried in a HCP-2 critical-point dryer (Hitachi, Tokyo, Japan) using CO2 and were sputter-coated with gold. Finally, the samples were visualized using a Hitachi S-450 SEM (Tokyo, Japan).

DNA quantification

The fresh liver and decellularized scaffolds ($n = 6$) were cut into small pieces and placed in centrifuge tubes, lyophilized later, and then DNA was isolated by using Genomic DNA Kit (TIANGEN Biotech, China) according to the manufacturer’s instructions. Briefly, samples were digested with DNA isolation solutions. Proteinase K digestion was used in the procedure and tubes containing the tissue samples were vortexed for 10 minutes and incubated at 56°C for 30 minutes. After the protein was precipitated and discarded, the remaining DNA was diluted with 1mL TE buffer (10 mM Tris-HCl, 1mM EDTA, pH 7.5). The total amount of DNA was quantified using Varioskan Flash (Thermo).

Histological evaluation of NAFLD/NASH model

The liver histology was evaluated by an expert pathologist who was blinded to the intervention condition. Histology was assessed using the NAFLD activity score (NAS), which computed from the grade of steatosis, lobular inflammation and hepatocellular ballooning. Specifically, the amount of steatosis (percentage of hepatocytes containing fat droplets) was scored as 0 (< 5%), 1 (5–33%), 2 (> 33–66%) and 3 (> 66%). Hepatocyte ballooning was classified as 0 (none), 1 (few) or 2 (many cells/ prominent ballooning). Foci of lobular inflammation were scored as 0 (no foci), 1 (< 2 foci per 200× field), 2 (2–4 foci per 200× field) and 3 (> 4 foci per 200× field). Fibrosis was scored as 0 (no fibrosis), 1 (perisinusoidal or portal/periportal fibrosis), 2 (perisinusoidal and portal/ periportal fibrosis), 3 (bridging fibrosis) and 4 (cirrhosis).

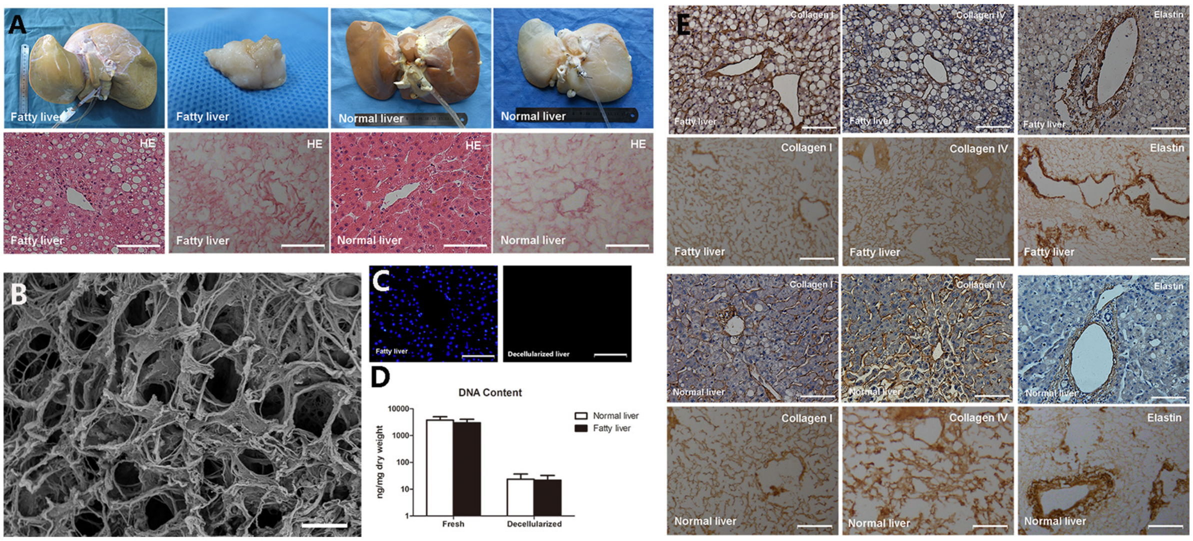

Supplementary Figure 1: Human liver decellularization and identify of its ECM. (**A**) Representative macroscopic images and hematoxylin and eosin (HE) staining of human fatty liver and normal liver before or after decellularization. The liver turned to be translucent with absence of cellular components in human fatty liver matrix (FLM) and human normal liver matrix (NLM). (**B**) Scanning electron microscopy (SEM) image of FLM showing the decellularized liver open spaces previously occupied by hepatocytes and other non-parenchymal cells. (**C**) The presence of intact nuclear material was evaluated by staining the fatty liver and FLM using DAPI. (**D**) DNA content of the fatty liver, normal liver, FLM and NLM (*n* = 6). (**E**) Comparison of ECM before and after decellularization, left to right: Collagen I, Collagen IV and elastin. Scale bars: 100 µm (A, C, E); 10 µm (B).

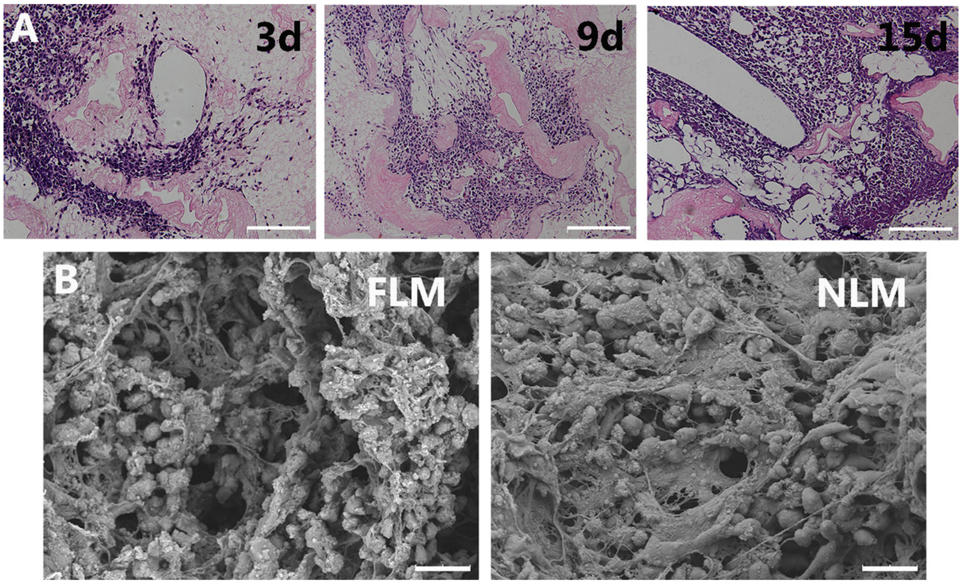

Supplementary Figure 2: HepG2 cells cultured in human decellularized liver matrix. (**A**) HE staining of the recellularized FLM, HepG2 cells proliferated well in the hFLM, left to right: 3d, 9d and 15d. (**B**) SEM images of HepG2 cells cultured in FLM and NLM at day 9. Scale bars: 200 μm (A); 20μm (B).

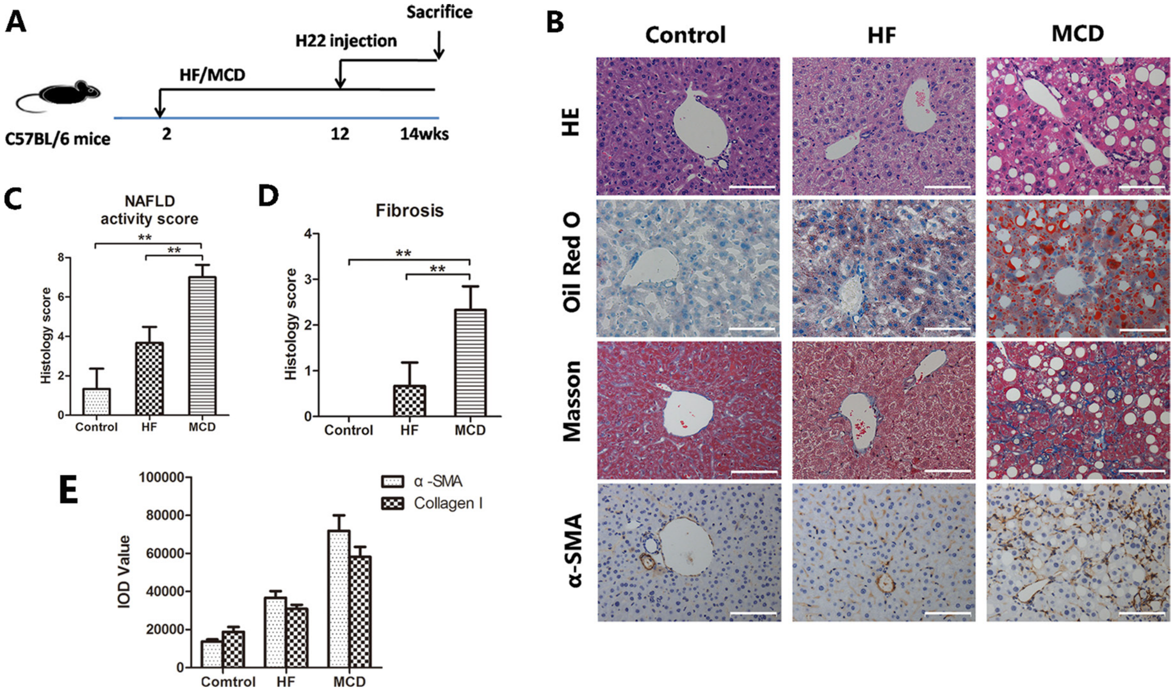

Supplementary Figure 3: Construction mouse models of NAFLD/NASH. (**A**) Experimental setup. (**B**) Histological analysis of pathological changes in different groups, left to right: HE, oil red O, Masson and α-SMA. Oil red O staining showed lipid droplets in the hepatocytes. Collagen fibrils could be seen in the sinusoids by Masson's trichrome staining and the activation of HSCs was confirmed by α-SMA staining. (**C**, **D**) NAFLD activity score consist of steatosis, Hepatocyte ballooning and lobular inflammation and fibrosis score were quantified ($n = 6$). (**E**) There was a close relationship between CAFs and Collagen I. Scale bars: 100 μm (B), $^{**}P < 0.001$.
